# Supplementary material for: Alopecia areata is not a risk factor for heart diseases: A 10-year retrospective cohort study
Source: PLoS One. 2021 May 7;16(5):e0250216. doi: 10.1371/journal.pone.0250216 (PMC8104430; doi:10.1371/journal.pone.0250216)
Supplement: Attachment — Submitted filename: Reponse to Reviewers.doc [file pone.0250216.s001.doc]

Editor-in-chief

March, 17th, 2021

Dear Editor,

Thank you for your decision regarding our manuscript.

After we have carefully considered the reviewer's comments, we have revised the manuscript accordingly. We hope that you find this revised version acceptable for publication in your distinguished journal.

Thank you for your valuable comments.

Sincerely yours,

Jee Woong Choi, M.D., Ph.D.

Professor

Department of Dermatology, Ajou University School of Medicine, Suwon, 16499, Korea

Tel: +82-31-219-5190

Fax: +82-31-219-5189

E-mail: dermaboy@gmail.com

**Reviewer comments:**

**Reviewer 1 #1:** A few clarifications: Were smoking/ substance abuse assessed as potential confounders. If potential confounders have been missed, add them as limitations.

**Authors’ response:**

- As the reviewer pointed out, smoking could affect the results. Therefore, we analyzed smoking related diseases, such as stress disorders, peripheral vascular diseases, atherosclerosis, and stroke, which could more directly affect heart diseases than smoking.
- Also, in Korea, substance abuse is very rare that we did not need to take consideration. Also, due to its rarity, we could not reach such information by Korean National Health Insurance Service-National Sample Cohort.

**Reviewer 1 #2:** The increased number of dyslipidemic patients in the AA group- whether on treatment, potential implications.

**Authors’ response:**

- As the reviewer pointed out, whether to be treated dyslipidemia could act as a bias. Thus, we adjusted such confounding factor ‘the presence of dyslipidemia’ when seeking hazard ratios, that we could be free from the possibility of the bias.

**Reviewer 1 #3:** The authors may discuss the mechanistics of AA and heart disease, a little more in depth (some authors have hinted at a benefit, as well).

**Authors’ response:**

- So far, the detailed mechanisms of alopecia areata are unclear. However, we could have been postulated that AA could be related to heart diseases by the various findings (Wang E.H., et al. Alopecia Areata is Associated with Increased Expression of Heart Disease Biomarker Cardiac Troponin I. Acta Derm Venereol. 2018;98(8):776-82; Wang E. et al. Development of autoimmune hair loss disease alopecia areata is associated with cardiac dysfunction in C3H/HeJ mice. PLoS One. 2013;8(4):e62935), and conducted this study. To deliver such perspective more clearly, we added this concept in the INTRODUCTION (Line 56-58)

**Reviewer 2 #1:** Unclear as to how ethnic differences would affect any potential association between AA and HD. The authors briefly mention this but do not elaborate.

**Authors’ response:**

- As we can see in other studies (Lane et al, Ethinic differences in blood pressure and the prevalence of hypertension in England. *Journal of Human Hypertension* 2002; 16: 267–273; Rampal et al, Ethinic differences in the prevalence of metabolic syndrome: Results from a multi-ethinic population-based survey in Malaysia, *Plos One*, 2012; 7(9): e46365), prevalence of various metabolic disease differs by the ethnicity. Because of the cultural and genetic differences, vulnerability to such diseases which are largely related to heart diseases could be varied by ethnicity.
- Thus, we were about to confirm the irrelevance between AA and cardiovascular disease, further general heart diseases by the large population of the Korean.
- To deliver our intention clearly, we inserted such contents in the INTRODUCTION. (line 61-62)

**Reviewer 2 #2:** Arbitrary definition of long-standing AA as greater than one year.

**Authors’ response:**

- As approximately 60% of AA patients have at least a partial hair regrowth by 1 year (according to Kang et al. Fitzpatrick’s Dermatology, 9^th^ edi.), previous studies (McDonald Hull et al. Guidelines for the management of alopecia areata. *British J Dermatol* 2003;149: 692–699; Pratt et al. Alopecia areata. *Nat Rev Dis Primers*. 2017; 3:17011) have used such criteria for ‘long-standing AA’. Thus, we defined ‘long-standing AA’ as more than a year, as well.

**Reviewer 2 #3:** Authors do not clarify why they chose a 1:5 control ratio and if a smaller (or larger) ratio would have had an impact on the findings.

**Authors’ response:**

- According to Hennessy et al. statistical power can be achieved when more than five controls per case were analyzed in case-control study design. (Hennessy et al. Factors Influencing the Optimal Control-to-Case Ratio in Matched Case-Control Studies. American Journal of Epid 1999; 149(2))

**Reviewer 2 #4:** Dyslipidemia was more common in AA group but it would have biased the results in favor of an association. Hence, this is likely not relevant.

**Authors’ response:**

- As the reviewer pointed out, whether to be treated dyslipidemia could act as a bias. Thus, we adjusted such confounding factor ‘the presence of dyslipidemia’ when seeking hazard ratios, that we could be free from the possibility of the bias.

**Reviewer 2 #5:** ICD code used for heart failure needs to be identified.

**Authors’ response:**

- Following this comment, we elaborated the ICD codes for heart diseases in METHODS (line 86-88).

**Reviewer 2 #6:** Chronic myocardial infarction is a poorly defined entity. I suspect the authors were referencing ICD I25.2 (old myocardial infarction).

**Authors’ response:**

- According to ICD-10 version of 2019, I25 code (chronic ischaemic heart disease) consists of I25.0 (Atherosclerotic cardiovascular disease, so described), I25.1 (Atherosclerotic heart disease), I25.2 (Old myocardial infarction), I25.3 (Aneurysm of heart), I25.4 (Coronary artery aneurysm and dissection), I25.5 (Ischaemic cardiomyopathy), I25.6 (Silent myocardial ischaemia), I25.8 (Other forms of chronic ischaemic heart disease), and I25.9 (Chronic ischaemic heart disease, unspecified)
- As those disease entities could be both a cause and effect of chronic myocardial infarction, we wanted to include all the subclassification codes in this study. To clarify this intention, we added the ICD-10 code description in METHODS. (line 86-88)
